# DNA topoisomerase IIα controls replication origin cluster licensing and firing time in Xenopus egg extracts

**DOI:** 10.1093/nar/gkt494

**Published:** 2013-06-11

**Authors:** Vincent Gaggioli, Barbara Le Viet, Thomas Germe, Olivier Hyrien

**Affiliations:** Institut de Biologie de l'Ecole Normale Supérieure (IBENS), S2-Génomique Fonctionnelle, CNRS UMR8197, Inserm U1024, 46 rue d'Ulm, 75005 Paris, France

## Abstract

Sperm chromatin incubated in Xenopus egg extracts undergoes origin licensing and nuclear assembly before DNA replication. We found that depletion of DNA topoisomerase IIα (topo IIα), the sole topo II isozyme of eggs and its inhibition by ICRF-193, which clamps topo IIα around DNA have opposite effects on these processes. ICRF-193 slowed down replication origin cluster activation and fork progression in a checkpoint-independent manner, without altering replicon size. In contrast, topo IIα depletion accelerated origin cluster activation, and topo IIα add-back negated overinitiation. Therefore, topo IIα is not required for DNA replication, but topo IIα clamps slow replication, probably by forming roadblocks. ICRF-193 had no effect on DNA synthesis when added after nuclear assembly, confirming that topo IIα activity is dispensable for replication and revealing that topo IIα clamps formed on replicating DNA do not block replication, presumably because topo IIα acts behind and not in front of forks. Topo IIα depletion increased, and topo IIα addition reduced, chromatin loading of MCM2-7 replicative helicase, whereas ICRF-193 did not affect MCM2-7 loading. Therefore, topo IIα restrains MCM2-7 loading in an ICRF-193-resistant manner during origin licensing, suggesting a model for establishing the sequential firing of origin clusters.

## INTRODUCTION

Eukaryotic DNA replication starts at multiple sites called replication origins (1). Origins tend to fire coordinately in clusters of 5–10 origins that are activated at different times through S phase (2). In mammalian cells, the replication timing program is established soon after mitosis, when chromosomal segments decondense and reposition in the early G1 nucleus (3). Early replication has been strongly correlated with transcriptional activity (4). Nevertheless, a replication timing program also exists in Xenopus egg extracts where no transcription is taking place (5). Despite their importance for embryonic development and genome stability, the mechanisms controlling the temporal programme of genome replication and the length of S phase have remained elusive. Recent experiments suggest that competition for limiting replication factors establishes the timing and efficiency of origin firing in fission yeast (6,7), budding yeast (8,9) and mammalian cells (10). The ability of individual origins to compete for limiting factors in S phase may rely on the relative amount of prereplicative complex (pre-RC) proteins ORC and MCMs loaded at each origin following mitosis (6,11). Alternatively, the Rif1 protein in fission yeast (12) and the Forkhead transcription factors Fkh1/2 in budding yeast (13) act as global regulators of origin firing time by affecting not pre-RC assembly but the loading of Cdc45, a cofactor of the MCM replicative helicase. Fkh1/2 exerts this effect in a transcription-independent manner, possibly by recruiting early origins into clusters where limiting replication factors are concentrated (13). Rif1 also regulates replication timing domains in human (14) and mouse (15) cells.

When demembranated Xenopus sperm nuclei are incubated in Xenopus egg extracts, the compact sperm chromatin decondenses, origins are ‘licensed’ for replication by binding of ORC and loading of MCM2-7 complexes and a nuclear envelope reforms. Following this ∼20 min nuclear assembly step, origins are activated, and the DNA is efficiently duplicated in ∼30 min (16). Origins fire throughout S phase as weakly synchronous clusters of 5–10 origins located at random sequences and spaced at 5–15 kb intervals (17–24). Pulse labeling of intranuclear replication foci revealed that these ∼1 Mb DNA domains replicate in a reproducible temporal sequence, as in somatic cells (5). However, within each ∼1 Mb domain, origins and origin clusters are activated in a random temporal order (5). The length of S phase can be extended by increasing the concentration of nuclei in egg extracts, which causes a slower activation of origin clusters without changing fork velocity or intracluster origin spacing (24,25). These results underscore the importance of staggered origin cluster activation in regulating S phase kinetics in egg extracts (24).

Chromatin further decondenses during S phase in a nuclear envelope-dependent manner in egg extracts (26). Interestingly, the catalytic DNA topoisomerase II (topo II) inhibitor ICRF-193 inhibits this nuclear envelope-dependent decondensation of chromatin and slows down S phase in egg extracts (27). Topo II is an ubiquitous and essential enzyme that has the unique ability to transport one double-stranded DNA segment through another. Topo II has multiple functions in the chromosome cycle, including the unlinking of replicating DNA (28,29). Topo IIα is the sole topo II isozyme expressed in egg extracts. Replication inhibition by ICRF-193 in egg extracts was proposed to result from a failure to unlink replicating DNA (27).

In principle, topo II can unlink replicating DNA by removing either (+) supercoils in front of the forks or (+) precatenanes (i.e. sister chromatid intertwinings) behind the forks (30). Positive supercoil removal seems to be a biologically important function of topo IIα because human topo IIα removes (+) supercoils faster than (−) supercoils in vitro (31). However, yeast and Drosophila topo II do not show this preference (32). Accumulation of (+) supercoils in the absence of topo II could conceivably impede replication fork progression, but topo I can remove (+) supercoils in the absence of topo II activity. Experiments with topo II inhibitors in Xenopus egg extracts showed that topo II can be trapped behind, but not in front of the forks, and unlinks replication intermediates in a non-redundant manner with topo I (33,34). These observations are consistent with precatenane, but not supercoil removal. Importantly, topo I cannot remove precatenanes. Precatenane accumulation in the absence of topo II activity would not be expected to impair replication fork progression. Furthermore, ICRF-193 traps topo II on the DNA in the form of a non-covalent intermediate named the closed clamp (35). It is therefore not clear whether the replication inhibition observed after ICRF-193 addition to egg extracts (27) is due to inhibition of DNA unlinking rather than some other effect of the closed clamps. For example, closed clamps trapped by ICRF-193 can perturb chromatin structure independently of topo IIα catalytic inhibition (36). In addition, ICRF-193 triggers sumoylation of topo II in human cells, which may also affect topo II function in DNA replication (37).

Here, we show that topo IIα depletion and inhibition with ICRF-193 have strikingly opposite effects on DNA replication in Xenopus egg extracts. Topo IIα depletion does not alter fork progression but accelerates DNA synthesis by increasing origin cluster activation. Topo IIα addition produces opposite effects to depletion. Topo IIα restrains MCM2-7 loading during origin licensing to establish a sequential firing of origin clusters. In contrast, ICRF-193 added during nuclear assembly slows origin cluster activation and fork progression without affecting MCM2-7 loading. ICRF-193 has no effect on DNA synthesis if added after nuclear assembly or if added to topo IIα-depleted extracts. We suggest that topo IIα clamps trapped by ICRF-193 during but not after nuclear assembly block replication because topo IIα acts on unreplicated DNA before S phase but behind the forks during S phase.

## MATERIALS AND METHODS

### Drugs

A 10 mM stock solution of ICRF-193 (Euromedex) and a 5 mM stock solution of Roscovitine (Sigma) were prepared in dimethylsulfoxide (Sigma) and stored in small aliquots at −20°C. Final working concentrations were 10 µM or 100 µM for ICRF-193 and 40 µM for Roscovitine. Caffeine (or buffer alone as control) was added to a final concentration of 5 mM from a 100 mM solution prepared as described (24).

### Replication of sperm nuclei in Xenopus egg extracts

Interphase egg extracts were prepared as described (38). Demembranated sperm nuclei were incubated at 1000 nuclei/μl in extracts supplemented with an energy regeneration mix [7.5 mM creatine phosphate, 1 mM ATP, 0.1 mM EGTA (pH 7.7), 1 mM MgCl_2_], creatine kinase (100 μg/ml) and cycloheximide (250 μg/ml). Experiments presented in Supplementary Figure S1 used other nuclei concentrations as indicated.

### Assay for topo II activity

Kinetoplast DNA (Inspiralis, Ltd, UK) was added to undiluted extract at a final concentration of 10 µg/ml and incubated at 23°C. At appropriate intervals, reactions were stopped by diluting aliquots in 1 volume of 2% SDS, 80 mM EDTA and 600 mM NaCl. Samples were digested with RNase A and proteinase K and electrophoresed on a 1% agarose gel in the presence of ethidium bromide. The gel was photographed under ultraviolet light, and the amount of decatenated DNA was measured using ImageJ.

### Quantitation of DNA synthesis

Demembranated sperm nuclei were incubated at 1000 nuclei/μl in extracts in the presence of 1/50 volume of [α^32^P]-dATP (3000 Ci/mol). Aliquots were diluted in 1 volume of 2% SDS, 80 mM EDTA, 600 mM NaCl, digested with RNase A and proteinase K and electrophoresed in 0.8% agarose. Gels were fixed in 7% trichloroacetic acid for 30 min, dried, exposed to phosphorimaging plates and revealed using a FLA-3000 phosphorimager (Fujifilm, Tokyo, Japan). Phosphorimager unit measurements of radiolabel incorporation were converted into standard radioactivity units using an internal standard to calculate the absolute amounts of replicated DNA as described (16). Experiments presented in Supplementary Figure S1 used other nuclei concentrations as indicated.

### Nascent DNA strand analysis by alkaline agarose gel electrophoresis

Demembranated sperm nuclei were incubated at 1000 nuclei/μl in extracts. Nascent strands were continuously labeled by adding 1/20 volume of [α-^32^P]-dATP (3000 Ci/mol) at the start of the incubation. For pulse-chase analysis, the label was added at 30 min and chased 2 min later with 2.5 mM dATP in the presence of 50 µM roscovitin (where indicated). Aliquots were stopped at the indicated time in 1 volume of 2% SDS, 80 mM EDTA, 600 mM NaCl. DNA was isolated using DNAZOL (Life Technologies, Inc.), according to the manufacturer’s protocol, taking care to minimize DNA strand breakage during sample preparation. The pellets were resuspended and electrophoresed on alkaline agarose gels (0.8% agarose, 50 mM NaOH, 2 mM EDTA) at 2 V/cm for 17 h at 4°C. Gels were fixed in 7% trichloroacetic acid for 30 min, dried, exposed to phosphorimaging plates and revealed using a Fluorescent Image Analyser FLA-3000 (Fujifilm, Tokyo, Japan).

### Fluorescent labeling of replicating nuclei

To visualize replication in individual nuclei, 33 μM rhodamine–dUTP (Roche Applied Science, Basel, Switzerland) was added at the indicated times for 2 or 5 min as indicated, and the reaction was stopped with 1×PBS and fixed in 4% p-formaldehyde. The reaction was spun through a 30% sucrose cushion in PBS on polylysine-coated glass coverslips. The coverslips were stained with 10 µg/ml Hoechst 33258 in 1 × PBS for 15 min, rinsed twice in 1 × PBS for 10 min and mounted in Vectashield. Images of nuclei were acquired using a 100 ×, 1.4 numerical aperture UPlanSApo objective on an Olympus IX 81 inverted microscope connected to a CoolSNAP HQ CCD camera (Photometrics, Tuscon, AZ, USA) run by MetaMorph version 6.3r7 (Molecular Devices, Union City, CA, USA). Images were processed with ImageJ (Rasband, 1997–2009). For analysis of replication foci, 30 Z stacks per nuclei were acquired at 0.2 μm interval. Images were processed in ImageJ using the Z projection function, convolution filter and a 2D iterative deconvolution function. Replication foci of at least 30 randomly chosen nuclei were counted on deconvoluted images after automated thresholding.

### DNA combing

DNA combing was performed as described (38) with slight modifications. Briefly, extracts were supplemented with 20 µM biotin–dUTP (Perkin Elmer, Waltham, MA, USA), and reactions were stopped at the indicated times with 10 volume ice-cold 1 × PBS. Labeled DNA was extracted, combed on silanized glass coverslips produced by liquid-phase silanization (39). The biotin label and total DNA were revealed with five layers of fluorochrome-conjugated antibodies: (i) Alexa Fluor 594 streptavidin (Molecular Probes, Invitrogen), (ii) biotinylated anti-streptavidin (Molecular Probes, Invitrogen) and mouse anti-DNA antibody (Abcyss MAB3034), (iii) Alexa Fluor 594 streptavidin and rabbit Alexa Fluor 488 anti-mouse antibody (Molecular Probes A11059), (iv) biotinylated anti-streptavidin and (v) Alexa Fluor 594 streptavidin and Alexa Fluor 488 goat anti-rabbit antibody (Molecular Probes A11008). Images of combed DNA were acquired using a Nikon Ti microscope with a 63× or a 100× oil immersion objective. The fields of view were chosen at random in the Alexa Fluor 488 channel and then photographed under the Alexa Fluor 488 and the Alexa Fluor 594 filter. Measurements on each molecule were made using ImageJ and compiled using Microsoft Excel (2004). Replication parameters are defined in the text as previously described (24).

### Immunodepletion and addition of topo II

Three different anti-Xenopus topo IIα antisera were used. One was a kind gift from D. Bogenhagen (40). Two other rabbit antisera were prepared against a 1:1 mixture of peptides CKQRDTKNTELQDLKR and KPVTYLEDSDDDF, corresponding to residues 1135–1150 and 1567–1579 of Xenopus topo IIα, respectively. Control antisera consisted of pre-immune sera from the same rabbits or a commercial non-immune rabbit sera (Sigma). Anti-Xenopus topo IIα antiserum or control serum were linked to protein A-sepharose fast flow beads (Amersham) according to a standard procedure (41). A volume of 150 µl extract or less was incubated with 150 µl of dried control or anti-topoIIα linked beads for 30 min at 4°C, and the procedure was repeated once.

For topo IIα addition experiments, 0.4 unit recombinant human topo IIα (USB) was added per microliters of extract, an amount equivalent to 2–3-fold the extract’s endogenous topo II activity, as judged by kinetoplast DNA decatenation. ICRF-193 (100 µM) fully inhibited the decatenation activity of the complemented extract (data not shown).

### Chromatin isolation and analysis

Demembranated sperm nuclei were incubated at 5000 nuclei/μl in extracts. Timed samples (20 µl) were diluted in 180 µl of ice cold Nuclear Isolation Buffer (50 mM KCl, 50 mM Hepes, 5 mM MgCl_2_, 2 mM DTT, 0.15 mM Spermin, 0.5 mM Spermidin, 1 µg/ml Leupeptin, 1 µg/ml Aprotinin, 1 µg/ml Pepstatin, 0.1% Triton X-100) containing 10% sucrose, slowly laid over 30% Sucrose-NIB cushions in 1.5 ml of Eppendorf tubes and centrifuged at 4°C, 4200 g for 5 min in a swinging-bucket rotor. The top layer was withdrawn, and the interface was rinsed twice with 200 µl of 10% Sucrose-NIB before recentrifuging at 6000*g* for 15 min. The chromatin pellet was resuspended in 20 µl of ice cold EB (5 mM KCL, 0.5 mM MgCl_2_, 0.2 mM DTT, 5 mM Hepes pH7.4), boiled for 5 min in the same volume of 2X Laemmli Buffer [4% SDS, 20% glycerol, 120 mM Tris–Cl (pH 6.8)] freshly added with 0.1 M DTT. In all, 6 µl aliquots were subjected to immunoblotting using 8% SDS–PAGE and ECL visualization (Thermo Scientific). The signals were quantified using ImageJ and normalized to histone H3 loading control.

## RESULTS

### ICRF-193 only perturbs DNA replication if added before S phase

**Figure 1.**
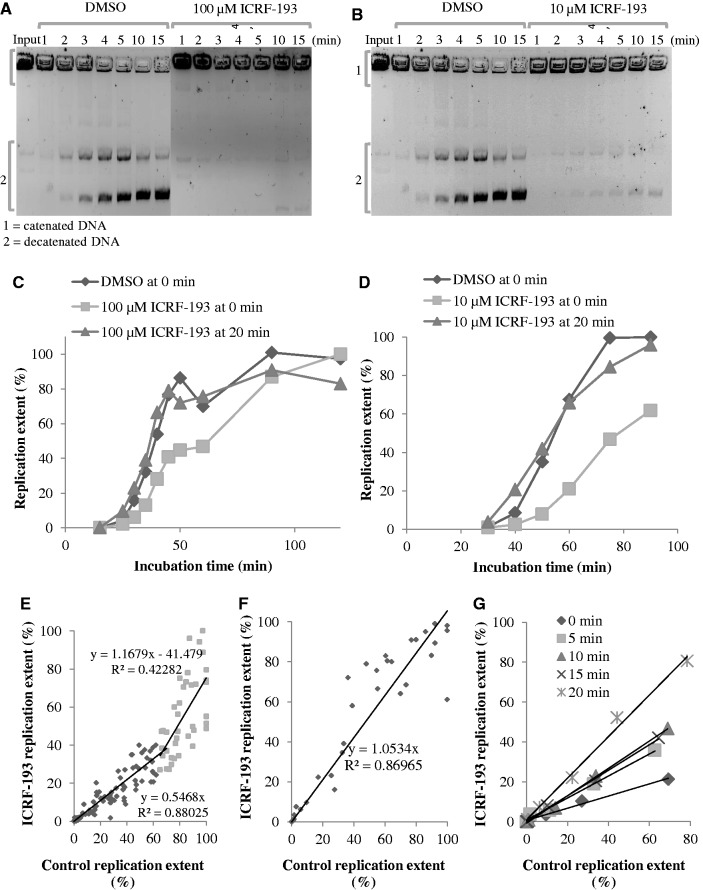
Concentration- and time-of-addition-dependent effects of ICRF-193 on DNA replication in egg extracts. (**A**) Kinetoplast DNA decatenation activity of egg extract in the presence of 100 µM ICRF-193, or in the presence of the drug solvent (DMSO) alone, was assessed at the indicated times. Input (1) and decatenated (2) DNA molecules are indicated. (**B**) Same experiment as in (A), but in the presence of 10 µM ICRF-193. (**C**) Sperm nuclei were incubated in egg extract in the presence of DMSO or 100 µM ICRF-193, added at 0 min or at 20 min. Replication was monitored by [α-^32^P]-dATP incorporation. (**D**) Same experiment as in C but using 10 µM ICRF-193. (**E**) Percentage of replication in the presence of 100 µM ICRF-193 added at 0 min plotted against the control. Data are from 14 independent experiments. (**F**) Same as in (E), but ICRF-193 was added at 20 min. Data are from six independent experiments. (**G**) Sperm nuclei were incubated in egg extract in the presence or absence of 100 µM ICRF-193 added at the times indicated by the symbols in the insert. The percentage of replication in the presence of the drug is plotted against the control.

The inhibition of Xenopus topo IIα activity by ICRF-193 was monitored by decatenation of kinetoplast DNA in Xenopus egg extracts. Decatenation was >95 and 100% inhibited by 10 µM and 100 µM ICRF-193, respectively (Figure 1A and B).

Addition of ICRF-193 (10 or 100 µM) to egg extracts at the same time as sperm nuclei (0 min) slowed incorporation of [α-^32^P]dATP into replicating nuclei. The start of DNA replication was not delayed, but its rate was reduced ∼2-fold and its completion delayed by ∼20 min (Figure 1C and D). The experiment was repeated 14 independent times using 1000 nuclei/µl and 100 µM ICRF-193 (Figure 1E). On average, replication was inhibited by 45% by the drug until the control reached 70% replication, then the control slowed down and the drug-treated extract caught up with it. Similar results were obtained using from 500 to 5000 nuclei/µl. Replication started later and progressed at a slower rate as the concentration of nuclei was increased, but ICRF-193 reduced the rate of replication ∼2-fold at all nuclei concentrations (Supplementary Figure S1).

When ICRF-193 was added at 20 min, after nuclear assembly had significantly progressed but before replication started, no inhibition of DNA replication was observed. This experiment was repeated six times using 1000 nuclei/µl with identical results (Figure 1F). Partial inhibition of replication was observed when ICRF-193 was added at 15 min, but maximal inhibition required addition at <5 min (Figure 1G). A control experiment verified that decatenation of a replicating plasmid was blocked by ICRF-193 added at 20 min as effectively as at 0 min (Supplementary Figure S2). We conclude that (i) topo IIα inhibition by ICRF-193 during S phase does not perturb DNA replication and (ii) topo IIα inhibition by ICRF-193 during nuclear assembly perturbs subsequent DNA replication.

### Prereplicative addition of ICRF-193 prolongs the lifetime of replication foci

**Figure 2.**
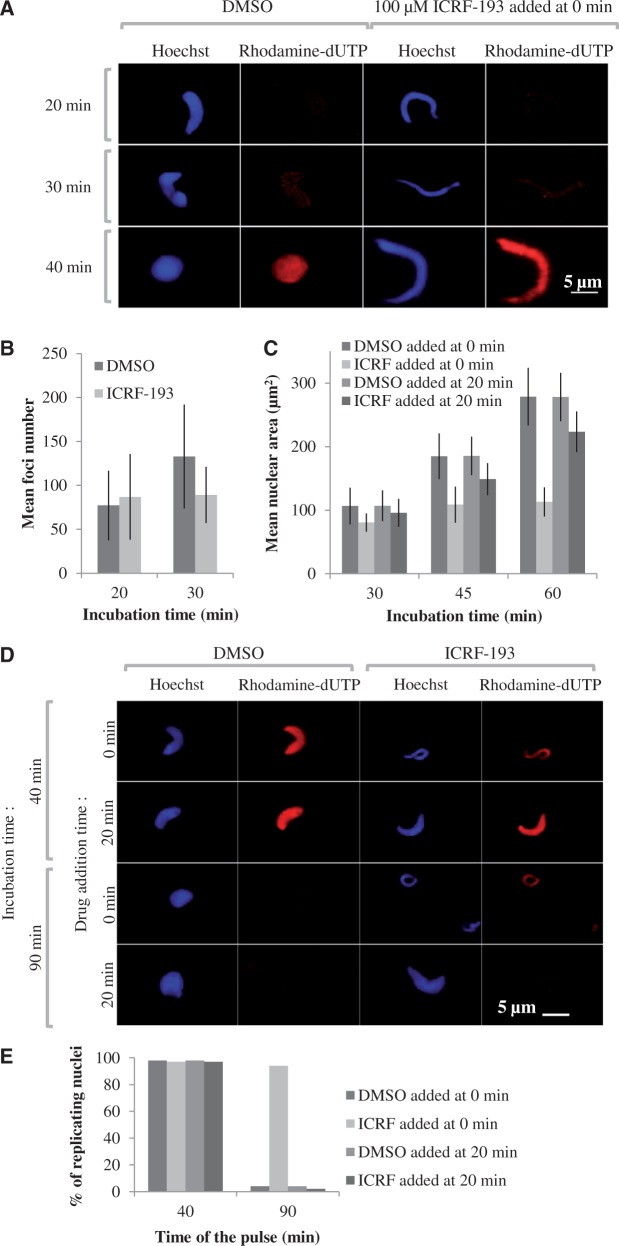
Time-of-addition-dependent effects of ICRF-193 on nuclear structure and replication foci. (**A**) Sperm nuclei were incubated in egg extract in the presence of 20 µM rhodamin–dUTP and either drug solvent alone (DMSO) or 100 µM ICRF-193 added at 0 min. Reactions were stopped at the indicated times, fixed and stained with Hoechst. Replication foci were imaged as described in the ‘Materials and Methods’ section. Scale bar = 5 µm. (**B**) Mean and standard deviation (*n* = 30) of the number of replication foci per nucleus at 20 min and 30 min with or without ICRF-193. (**C**) Mean ± SD (*n* = 50) of nuclear area at 30, 45 and 60 min with DMSO or ICRF-193 added at 0 or 20 min. (**D**) Replication run-on assay. Sperm nuclei in egg extracts were pulsed for 5 min with rhodamin–dUTP at 40 or 90 min, fixed and stained with Hoechst. (**E**) Percentage of replicating nuclei at 40 or 90 min in the experiment shown in (D). In all, 150–200 nuclei were counted per sample.

Sperm nuclei decondensation and replication in the presence or absence of ICRF-193 were monitored by fluorescence microscopy (Figure 2). Sperm nuclei acquired a rounded shape in control extracts but remained elongated when the drug was added at 0 min (Figure 2A). No change in the appearance or mean number of replication foci was observed in the presence of the drug when nuclei were pulse-labeled with rhodamin–dUTP for 2 min at 20 min and 30 min (Figure 2A and B). The mean area of individual nuclei was not significantly affected by ICRF-193 until 30 min but then failed to increase as in the control, showing that the nuclear envelope-dependent decondensation of chromatin was perturbed (Figure 2C), as previously observed (27). Importantly, this perturbation of decondensation was minimized when ICRF-193 was added at 20 min (Figure 2C).

To monitor completion of S phase, nuclei were pulse-labeled with rhodamin–dUTP for 5 min at 40 and 90 min (Figure 2D and E). Replication was still going on at 40 min but had completed at 90 min in the control or when ICRF-193 was added at 20 min. When ICRF-193 was added at 0 min, however, 98% of the pulsed nuclei were still homogeneously labeled at 90 min. We conclude that ICRF-193, when added at 0 min, does not perturb entry into S phase but strongly delays completion of DNA replication in each nucleus and in most if not all replication foci. In contrast, adding ICRF-193 at 20 min did not significantly delay replication completion.

### ICRF-193 affects both DNA replication initiation and elongation

**Figure 3.**
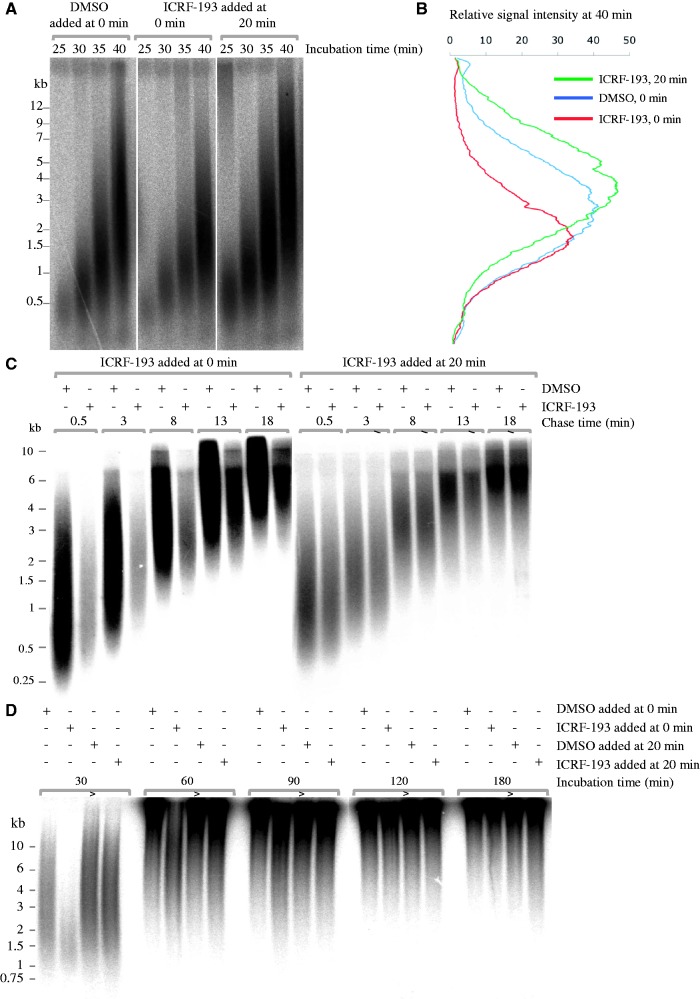
Alkaline gel electrophoretic analysis of ICRF-193-induced perturbation of DNA synthesis. (**A**) ICRF-193 added at 0 min, but not 20 min, reduces the abundance and perturbs the growth of nascent DNA strands. Sperm nuclei were incubated in egg extract containing [α-^32^P]-dATP in the presence of DMSO or 100 µM ICRF-193, added at 0 or at 20 min. Nascent strand abundance and growth was monitored by alkaline gel electrophoresis at the indicated time points. (**B**) Phosphorimager scan profiles of the 40 min lanes in (A). (**C**) Pulse-chase analysis of nascent strand growth. Sperm nuclei incubated in egg extract in the presence of DMSO or ICRF-193 added at 0 min or 20 min as indicated were labeled at 28 min with a 2 min pulse of [α-^32^P]dATP and chased for the indicated times with unlabeled dATP in the presence of roscovitin. Nascent strands were analyzed as in (A). (**D**) Nascent strand maturation analysis. Same experiment as in (A) except for the indicated times.

To further understand the mechanism(s) by which ICRF-193 reduces DNA synthesis, sperm nuclei were incubated in extracts with [α-^32^P]dATP, and nascent DNA strands were analyzed at 25, 30, 35 and 40 min by alkaline agarose gel electrophoresis (Figure 3A and B). ICRF-193 added at 0 min did not delay the start of S phase but reduced the incorporation of radioactive label by ∼40% at all time points. The growth of nascent strands was delayed with respect to the control when ICRF-193 was added at 0 min, but not 20 min. Strand size distributions depend on initiation, elongation and termination rates. Elongation rates were measured by labeling nascent strands at 28 min by a 2-min pulse of [α-^32^P]dATP, chasing the label with unlabeled dATP in the presence of roscovitin, a cyclin-dependent kinase inhibitor that blocks further initiation, and measuring strand growth between early time points, when replicon merge was minimal (Figure 3C). Elongation rates were found to be 500 nt.min^−^^1^ in the control but 400 nt.min^−^^1^ when ICRF-193 was added at 0 min. This 20% inhibition did not fully account for the 40% inhibition of [α-^32^P]dATP incorporation, suggesting that origin firing was also reduced, as shown below by DNA combing.

To determine whether a slower merge of replicons could contribute to the slower growth of nascent strands later in S phase, the accumulation of high-molecular weight (HMW) nascent strands was monitored up to 180 min (Figure 3D). HMW material started to accumulate in the wells of the gel at 60 min in the controls or when ICRF-193 was added at 20 min. When ICRF-193 was added at 0 min, HMW strands only accumulated at 90 min, but strand size distribution became identical to controls at 120 min. No accumulation of replicon-sized strands was detected, arguing against a prolonged block of replicon merge. The slower initiation and elongation rates observed when ICRF-193 was added at 0 min were sufficient to explain the delayed accumulation of HMW strands.

Nascent strand growth and abundance were unaffected when the drug was added at 20 min. Therefore, topo II activity appears dispensable during DNA replication for normal rates of origin firing and fork progression. This conclusion was confirmed by immunodepletion of topo IIα (see later in the text). Overall, these results imply that topo IIα clamps trapped by ICRF-193 at 0 min, but not at 20 min, slowed down replication fork establishment and progression (see ‘Discussion’ section).

### ICRF-193 delays the firing of entire origin clusters but does not affect replicon size

**Figure 4.**
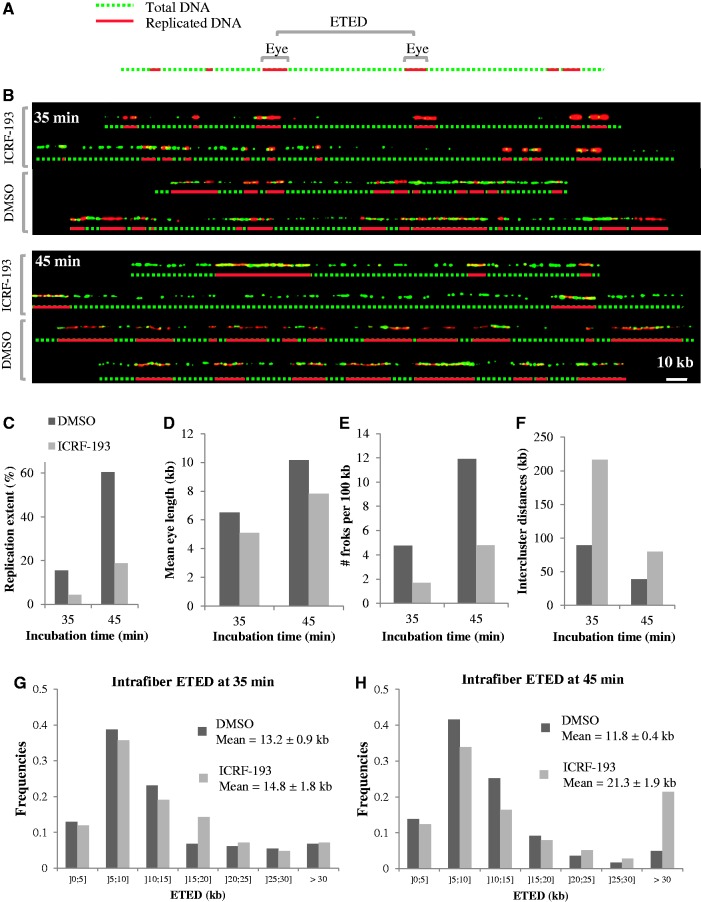
DNA combing analysis of ICRF-193-induced perturbation of DNA synthesis. Sperm nuclei were incubated in egg extract plus biotin–dUTP for 35 or 45 min. DNA was extracted, combed on silanized coverslips and biotin-labeled replication eyes and total DNA were revealed using red and green fluorochrome-conjugated antibodies, respectively. (**A**) An exemplary DNA fiber visualized in the two channels and a merge picture. The midpoints of consecutive eyes are indicated by vertical white arrows. The distance between two consecutive arrows is referred to as eye-to-eye distance (ETED). (**B**) Representative single DNA molecules from the indicated samples and interpretative diagrams. Scale bar, 10 kb. Total replication extent (**C**), mean eye length (**D**), total fork density (**E**), intercluster distances (**F**) and distributions of ETED (size bins indicated in kb below horizontal axis) at 35 (**G**) and 45 min (**H**) were measured for each condition as described in the text.

We used DNA combing (21,38) to further understand the effects of ICRF-193 (added at 0 min) on DNA replication. Sperm nuclei were incubated in egg extracts with biotin–dUTP. At 35 and 45 min, DNA was purified and combed. Biotin-labeled replication eyes were stained in red, and total DNA was stained in green (Figure 4A and B). Total replication extent, defined by the length ratio of all biotin stretches to total DNA, was reduced ∼3-fold by ICRF-193 (4.3 versus 15.4% at 35 min and 18.7 versus 60.4% at 45 min; Figure 4C), consistent with the inhibition observed by [α-^32^P]dATP incorporation. Replication inhibition was explained by a ∼22% reduction in mean eye length (Figure 4D; 5.1 versus 6.5 kb at 35 min; 7.8 versus 10.2 kb at 45 min) and a ∼62% reduction in global fork density (Figure 4E; 1.7 versus 4.75 forks/100 kb of total DNA at 35 min; 4.78 versus 11.9 forks/100 kb of total DNA at 45 min), in agreement with gel electrophoresis measurements of nascent strand size and abundance (Figure 3). However, at comparable fiber replication extent (18.7% at 45 min with ICRF-193 versus 15.4% at 35 min in the control), fork densities (4.78 versus 4.75 forks/100 kb of total DNA) and eye lengths (7.8 versus 6.5 kb) were similar. These results show that ICRF-193 strongly delayed but did not block origin activation.

As combed DNA fibers are of finite size, the center–center distances between consecutive eyes (eye-to-eye distances) are biased against large interorigin and intercluster distances. However, the mean of the missing distances from the distribution (i.e. essentially the mean intercluster distance) can be computed by comparing the global fork density (which is twice the inverse of the true mean eye-to-eye distance) with the local eye-to-eye distances measured on single fibers, as detailed elsewhere (24). Thus, in each sample, the global eye-to-eye distance calculated from the fork density was much larger than the mean local (intrafiber) eye-to-eye distance, owing to exclusion of intercluster distances, and the discrepancy was stronger in the presence of the drug (Figure 4E, G and H; control, 42.1 versus 13.2 kb at 35 min and 16.8 versus 11.8 kb at 45 min; with drug, 117.4 versus 14.8 kb at 35 min and 41.9 versus 21.3 kb at 45 min). The computed intercluster distances (Figure 4F) were 2–3-fold larger in the presence of the drug than in the control at 35 min (216.4 versus 89.3 kb) and 45 min (79.6 versus 38.6 kb). However, they were similar when drug-treated and control samples of similar replication extent were compared (79.6 versus 89.3 kb). These results suggest that ICRF-193 delayed but did not block the activation of late origin clusters. The intrafiber eye-to-eye distances at 35 and 45 min with or without the drug all peaked at ∼10 kb (Figure 4G and H) and were similarly spread around the peak save for a higher fraction of >30 kb distances with ICRF-193. Together, these results show that ICRF-193 slowed down the activation of origin clusters but did not affect interorigin distances within clusters. Once a cluster was activated, ICRF-193 no longer perturbed initiation within it.

### The replication checkpoint does not mediate the effects of ICRF-193

Replication fork stalling agents can activate a checkpoint that blocks further initiation until the stall is removed (42). Checkpoint inhibition in the presence of such agents can allow the recruitment of otherwise ‘dormant’ origins, resulting in replication rescue (43). The Chk1 kinase, generally considered as the main downstream effector of the ATR checkpoint kinase (44), was reported to remain unphosphorylated in the presence of ICRF-193 in Xenopus egg extracts (45). However, it is controversial whether Chk1 is a downstream effector of the replication checkpoint in Xenopus (46,47).

To address whether downregulation of origin clusters by ICRF-193 results from checkpoint activation owing to fork slowing, sperm nuclei were replicated in the presence or absence of ICRF-193 plus or minus caffeine, an inhibitor of ATM/ATR checkpoint kinases (Supplementary Figure S3). Caffeine alone can stimulate DNA replication (24,47) by reducing both interorigin and intercluster distances (24), suggesting checkpoint control of origin firing even in absence of exogenous replication inhibitors. This response to caffeine was found here to vary between independently prepared extracts (Supplementary Figure S3). Five of eight extracts responded strongly, one responded weakly and two others did not respond at all. Caffeine failed to rescue replication inhibition by ICRF-193 in the latter three extracts. Caffeine rescued ICRF-193 inhibition in the five other extracts but to an intermediate level that never matched the kinetics observed in the presence of caffeine alone (Supplementary Figure S3). We conclude that the basal activity of the replication checkpoint varies between extracts, and that its inhibition can activate dormant origins to a consistent level, in the presence or absence of ICRF-193, but that ICRF-193 by itself does not modulate this basal checkpoint activity.

We also observed that caffeine neither rescued nor exacerbated the slower growth of nascent strands induced by ICRF-193, even when caffeine did stimulate origins (Supplementary Figure S4). This confirms that the effect of ICRF-193 on replication fork progression is not a consequence of checkpoint induction.

### Topo II depletion has opposite effects to ICRF-193 on DNA replication

**Figure 5.**
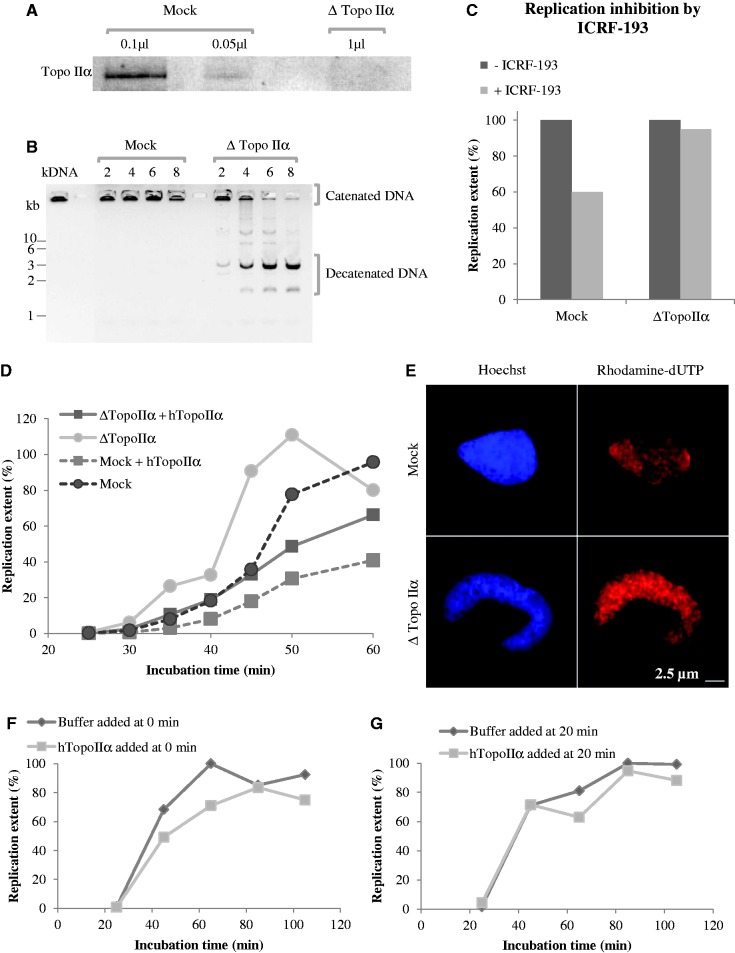
Effects of topo IIα depletion and addition on DNA replication kinetics and nuclear structure. (**A** and **B**) Topo IIα immunodepletion. Mock-depleted and topo IIα-depleted extracts were analyzed by western blotting with an anti-Xtopo IIα antibody (A; volume of extracts are indicated) and by kDNA decatenation (B). (**C**) Sperm nuclei were incubated in mock-depleted or topoIIα-depleted extracts containing [α-^32^P]-dATP plus or minus 100 µM ICRF-193 for 60 min, and the ratio of replicated DNA with and without ICRF-193 was calculated. (**D**) Sperm nuclei were incubated in mock-depleted (dotted lines) or topo IIα-depleted (solid lines) extracts containing [α-^32^P]-dATP supplemented with buffer (circles) or with recombinant human topo IIα (squares), and replication efficiency was measured at the indicated times. (**E**) Sperm nuclei were incubated in mock-depleted (upper panels) or topo IIα-depleted (lower panels) extracts in the presence of rhodamin–dUTP for 90 min, fixed and stained with Hoechst and observed under a fluorescence microscope. Scale bar, 2.5 µm. (**F**) Sperm nuclei were incubated in extracts containing [α-^32^P]-dATP supplemented with buffer (circles) or with recombinant human topo IIα (squares) added at 0 min, and replication was measured at the indicated times. (**G**) Same as in (F), except that buffer or topo IIα were added at 20 min.

The prereplicative effects of ICRF-193 could result from catalytic inhibition of topo IIα or from trapping topo IIα clamps on chromatin or perhaps from some other mechanism unrelated to topo IIα inhibition. To address these questions, we examined the effects of topo IIα immunodepletion on sperm chromatin decondensation and replication. The extent of topo IIα immunodepletion was estimated to >95% by western blot (Figure 5A) and >99% by kDNA decatenation (Figure 5B). Addition of ICRF-193 to mock-depleted extracts inhibited DNA replication as with undepleted extracts. However, topo IIα depletion completely abolished the effect of ICRF-193 on DNA replication as judged by [α-^32^P]dATP incorporation (Figure 5C). This result shows that ICRF-193 has no detectable effect on DNA replication other than by topo IIα inhibition.

Strikingly, topo IIα depletion accelerated DNA replication ∼2-fold with respect to mock depletion (Figure 5D). This surprising result was reproduced seven times using three different anti-topo IIα antisera and four different non-immune control sera. The stimulation of DNA replication was negated by re-addition of recombinant human topo IIα (htopo IIα) (Figure 5D; reproduced three times). These results show that catalytic inhibition of topo II cannot be the mechanism by which ICRF-193 slows down replication. Instead, the effect of ICRF-193 must only result from the trapping of topo IIα in the form of closed protein clamps on the DNA. Interestingly, addition of purified topo IIα at 0 min to mock-depleted (Figure 5D) or undepleted (Figure 5F) extracts slowed down replication. However, addition of topo IIα at 20 min did not slow down replication (Figure 5G), reminiscent of the ICRF-193 addition time effects (Figure 1). These results suggest that topo IIα is normally limiting for establishing replication timing during nuclear assembly and raise the possibility that ICRF-193 enhances this reprogramming action by stabilizing topo IIα association with chromatin (but see next section).

Topo IIα immunodepletion blocked the nuclear envelope-dependent decondensation of chromatin just as well as ICRF-193 (Figure 5E), suggesting that chromatin decondensation is not limiting for rapid DNA replication, and that slower decondensation is not the cause for slower replication in the presence of ICRF-193.

**Figure 6.**
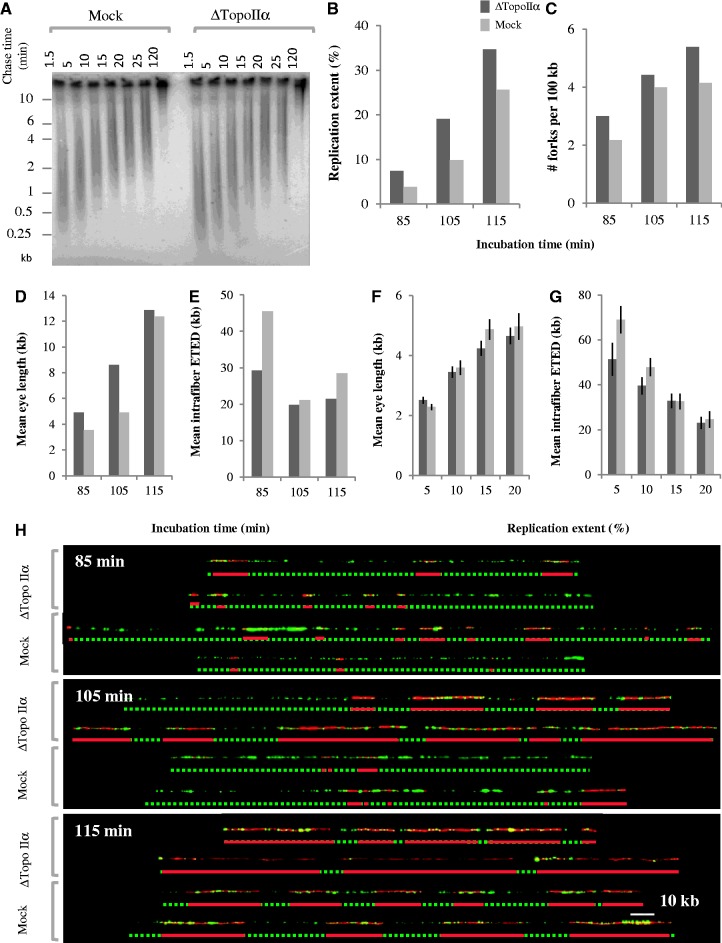
Gel electrophoretic and DNA combing analysis of replication intermediates synthesized in mock- and topoIIα-depleted extracts. (**A**) Pulse-chase analysis of nascent strand growth. Sperm nuclei incubated in mock- or topoIIα-depleted extracts were labeled at 50 min with a 10-min pulse of [α-^32^P]dATP and chased for the indicated times with unlabeled dATP in the presence of roscovitin. Nascent strands were electrophoresed in an alkaline agarose gel as in Figure 3A. (B–H) DNA combing analysis. Sperm nuclei were incubated in egg extract plus biotin–dUTP for 85, 105 or 115 min. DNA was extracted and combed as described in Figure 4. Total replication extent (**B**), total fork density (**C**), overall eye length (**D**) and mean intrafiber eye-to-eye distances (**E**) are shown for the three time points. To compare replication patterns within clusters, fibers subsets of comparable replication extents were selected and intrafiber eye lengths (**F**) and eye-to-eye distances (**G**) were measured. (**H**) Representative single DNA molecules from the indicated time samples and interpretative diagrams. Scale bar, 10 kb.

The role of topo IIα in orchestrating S phase was further investigated by analyzing replication intermediates synthesized in topo IIα-depleted extracts (Figure 6). Pulse-chase experiments (Figure 6A) showed that topo IIα depletion did not increase the rate of nascent strand elongation with respect to mock depletion. In contrast, DNA combing (Figure 6B–G) revealed that replicating DNA had more numerous and larger replication eyes in topo IIα-depleted than in mock-depleted extracts at the same time points. Replication extents at 85, 105 and 115 min were 40–90% higher in topo IIα-depleted extract than in the same extract depleted with mock antibodies (Figure 6B), consistent with [α-^32^P]dATP incorporation experiments (Figure 5D). The immunodepletion protocol can non-specifically reduce extract activity to a variable degree. Thus, the extract used in Figure 6B happened to be slower than in Figure 5D. Nevertheless, topo IIα-depleted extracts were always faster than their matched mock-depleted extracts. The faster replication in topo IIα-depleted than mock-depleted extract arose from a ∼30% increase in fork density (Figure 6C) and a ∼40% increase in replication eye length (Figure 6D), suggesting faster origin activation. As S phase progressed, however, the intrafiber eye-to-eye distance reached the same minimum in both samples (Figure 6E), and when fibers of similar replication extent and size distributions were compared, eye lengths (Figure 6F) and eye-to-eye distances (Figure 6G) were not significantly different in topo IIα- and mock-depleted extracts. Together, these results suggest that in the absence of topo IIα the replication of some clusters is advanced with respect to mock depletion, but once these regions start replicating, the pattern of origin activation is the same as in mock-depleted extracts. Importantly, the addition of purified topo IIα negated the effects of topo IIα depletion (Figure 5D, and data not shown). These results show that topo IIα is directly involved in a mechanism that delays the firing of some origin clusters.

### Effect of topo II depletion or addition or inhibition on replication licensing

**Figure 7.**
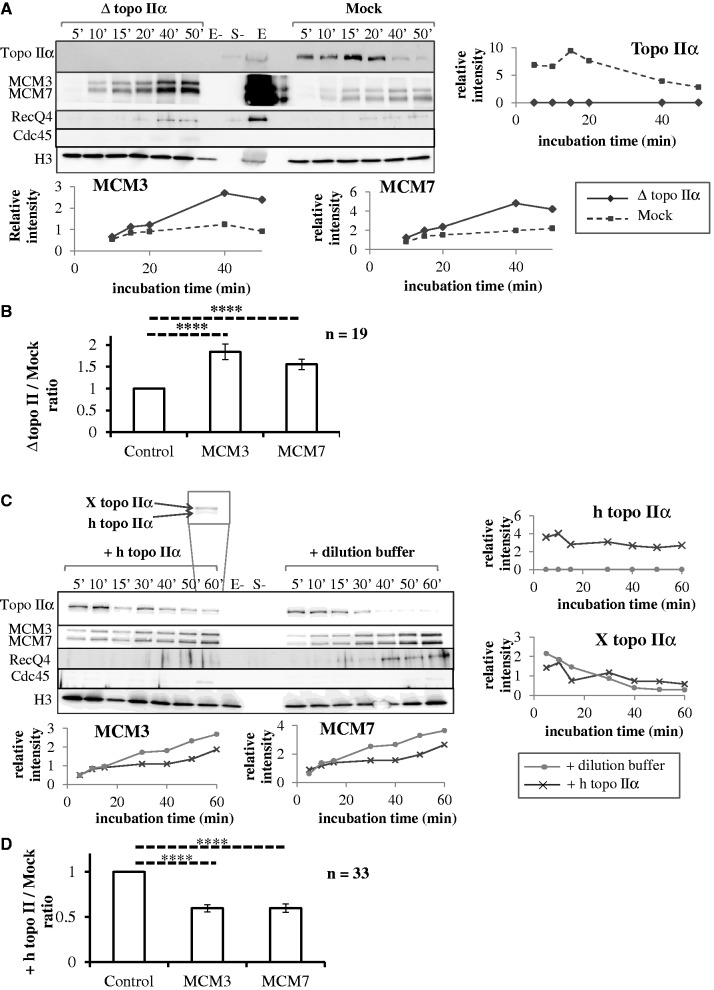
Effects of topo IIα depletion (**A** and **B**) or addition (**C** and **D**) on chromatin binding of MCM3, MCM7, RecQ4, Cdc45 and topo IIα. Sperm nuclei were incubated in mock-depleted (A, dotted lines), topo IIα-depleted (A, solid lines) or undepleted (C) extracts added with recombinant htopo IIα (C, dark gray lines) or htopo IIα dilution buffer (C, light gray lines). (A and C) Purified chromatin at indicated times was analyzed by western blotting for the indicated proteins. Histone H3 was used as a loading control. Samples without egg extract (E-) or without sperm nuclei (S-) and 3 µl of unprocessed extract (**E**) were loaded as internal controls. The inset shown in (C) illustrates the electrophoretic resolution of human and Xenopus topo IIα. The signals quantified using Image J and normalized to histone H3 are shown as graphs. (B and D) Quantitation of MCM loading over multiple experiments and time points. Shown is the average ratio ± S.E.M of MCM signal (normalized to histone H3), in topo IIα-depleted (B) or htopo IIα-added (D) extracts, to MCM signal in control extracts. The observed ratios are significantly (****P < 0.10^−4^) different from the expected value of 1 in the null hypothesis, as indicated by asterisks and dotted lines.

**Figure 8.**
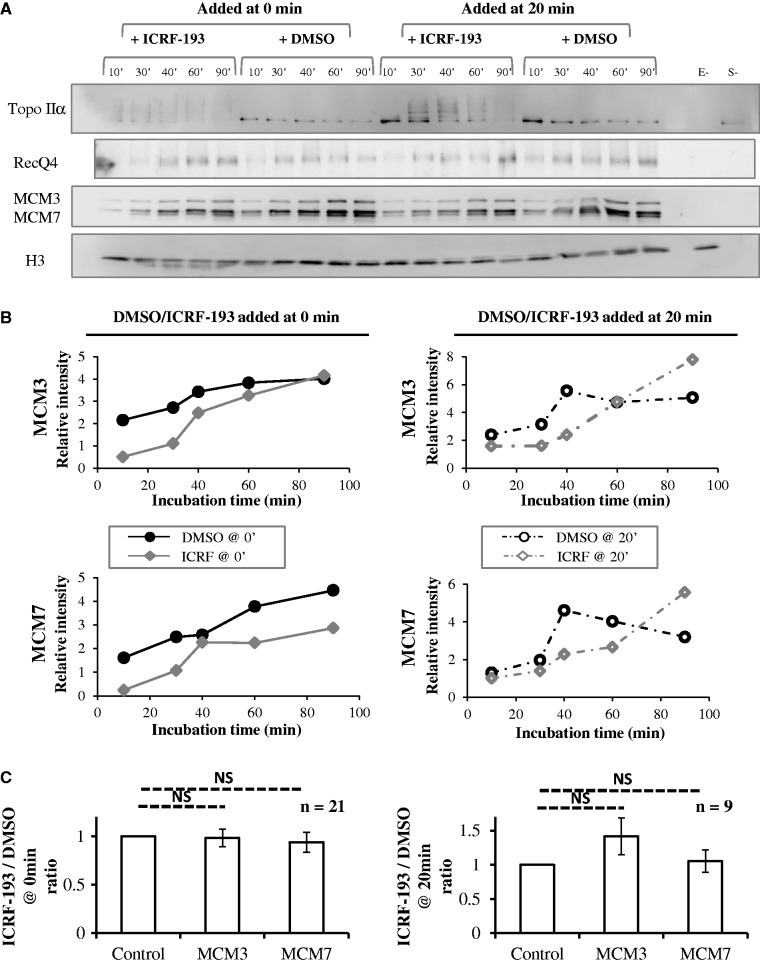
Effects of topo IIα inhibition by ICRF-193 on chromatin binding of MCM3, MCM7, RecQ4 and topo IIα. Sperm nuclei were incubated in undepleted extracts added with DMSO or 100 µM ICRF-193 at 0 or 20 min. (**A**) Purified chromatin at indicated times was analyzed by western blotting for the indicated proteins. Histone H3 was used as a loading control. Samples without egg extract (E-) or without sperm nuclei (S-) and 3 µl of unprocessed extract (E) were loaded as internal controls. (**B**) The signals quantified using Image J and normalized to histone H3 are shown as graphs. (**C**) Quantitation of MCM loading over multiple experiments and time points. Shown is the average ratio ± SEM of MCM signal (normalized to histone H3) in ICRF-193-treated extracts to MCM signal in control extracts. The observed ratios are not significantly different from the expected value of 1 in the null hypothesis.

To further understand the role of topoIIα on origin firing time regulation, the binding of topo IIα to sperm chromatin and the effects of topo IIα depletion (Figure 7A and B) or addition (Figure 7C and D) or inhibition (Figure 8) on the chromatin binding of topo IIα, MCM3, MCM7, RecQ4 and Cdc45 proteins were analyzed by western blot. A high concentration of sperm nuclei (5000/µl) was used in these experiments. This improved the detection of chromatin-bound proteins but slowed down replication which started at ∼60 min in undepleted extracts (Supplementary Figure S1) or even later in depleted extracts (not shown).

Topo IIα rapidly bound to chromatin at the start of the incubation and progressively dissociated thereafter (Figures 7A and C and 8A). Added htopo IIα followed the same behavior and did not alter the behavior of endogenous topo IIα (Figure 7C). No topo IIα was associated with sperm nuclei before incubation in egg extracts (Figures 8A and C and 9A lanes E-) or after incubation in topo IIα-depleted extracts (Figure 7A). Importantly, topo IIα depletion resulted in a ∼2-fold increase in MCM3 and MCM7 chromatin loading (Figure 7A), and topo IIα addition had the opposite effect (Figure 7C). No significant Cdc45 binding was detected before 50–60 min (Figure 7A and C), confirming that the observed changes in the level of MCM binding occurred during and not after origin licensing. These two experiments were repeated with three (depletion) and four (addition) independently prepared extracts (19 and 33 time points, respectively). On average, the loading of MCM3 and MCM7 was increased 1.84 ± 0.18-fold (*P* < 10^−^^4^) and 1.55 ± 0.12-fold (*P* < 10^−^^4^), respectively, on topo IIα depletion. On the other hand, on htopo IIα addition, the loading of MCM3 and MCM7 was reduced to 0.59 ± 0.04-fold (*P* < 10^−^^4^) and 0.60 ± 0.05-fold (*P* < 10^−^^4^), respectively, that of the control. An increase/decrease of ORC2 binding on topo IIα depletion/addition was also observed but more variable (not shown). Overall, these results show that topo IIα rapidly binds chromatin to negatively regulate origin licensing during nuclear assembly and then dissociates when licensing is complete. We propose that the increased MCM loading explains the faster activation of origin clusters and acceleration of S phase observed after topo IIα depletion.

To gain further insight into the activation of preRCs, we monitored the binding of RecQ4, a putative Sld2 homolog required for replication. Chromatin binding of RecQ4 was detected before the binding of Cdc45 and the onset of DNA replication, as reported previously (48,49). Topo IIα depletion increased RecQ4 binding, and topo IIα addition had the opposite effect. These observations are consistent with the possibility that alterations of MCM loading owing to manipulation of topo IIα levels proportionately impact the binding of RecQ4. However, it has been debated whether preRC is required for RecQ4 binding (48,49). We do not exclude the possibility that RecQ4 binding is affected by topo IIα addition or depletion independently of MCM loading.

We considered that ICRF-193 may mimic the effects of excess topo IIα addition by stabilizing topo IIα on chromatin. Chromatin binding of MCM3 and MCM7 proteins was investigated in the presence or absence of ICRF-193 added at 0 min (Figure 8A and B left). This experiment was repeated with four independently prepared extracts (total 21 time points). Although some decrease in MCM3 and MCM7 binding was observed in the presence of ICRF-193 in the experiment shown on Figure 8A and B, a slight increase was observed in the three other experiments. On average, the variation was non-significant, 0.98 ± 0.09-fold (*P* = 0.85) and 0.94 ± 0.10-fold (*P* = 0.54) for MCM3 and MCM7, respectively (Figure 8C, left). We neither observed a consistent effect of ICRF-193 addition at 20 min. On average, the variation was non-significant, 1.42 ± 0.27-fold (*P* = 0.12) for MCM3 and 1.05 ± 0.16-fold (*P* = 0.74) for MCM7 (Figure 8C, right). We conclude that in contrast to topo IIα addition or depletion, inhibition of topo IIα with ICRF-193 does not reproducibly affect MCM loading. These results imply that topo IIα regulates origin licensing in an ICRF-193-resistant manner and that ICRF-193 exerts its DNA replication inhibitory effect independently of origin licensing, probably through a roadblock effect of topo IIα clamps on establishment and progression of replication forks.

ICRF-193 added at 0 min slightly reduced the binding of RecQ4 at 10–30 min but not later (Figure 8A). Thus, similar amounts of RecQ4 were bound to chromatin before replication onset in the presence or absence of ICRF-193. Although Cdc45 binding was not detected here, we found in other experiments (Supplementary Figure S6) that when ICRF-193 was added at 0 min, the chromatin binding of Cdc45 was not affected, but its dissociation during the progression of DNA replication was slower, consistent with the prolonged S phase observed in these conditions. These results suggest that neither RecQ4 nor Cdc45 binding to chromatin are limiting for DNA replication after addition of ICRF-193 at 0 min. Other events such as helicase progression on unreplicated DNA must be affected by topo IIα clamps.

To explain the time-of-addition effects of ICRF-193 on DNA replication, we considered that ICRF-193 may trap a higher amount of topo IIα clamps when added at 0 min than at 20 min, owing to the dissociation of most topo IIα from chromatin before S phase entry. Instead, a lower amount of topo IIα was bound to chromatin when ICRF-193 was added at 0 min rather than at 20 min (Figure 8A). Immediately after ICRF-193 addition, topo IIα formed a ladder of higher molecular weight species presumably owing to its sumoylation, as reported in human cells (37). These results suggest that the time-of-addition effects of ICRF-193 on DNA replication are unlikely to result from different amounts of closed clamps or different extents of topo IIα modification. We suggest that topo IIα clamps trapped at 0 min and 20 min differ in their ability to block replication because topo IIα acts on unreplicated DNA during nuclear assembly but is targeted to replicated sisters during S phase (33,50) (see ‘Discussion’ section).

## DISCUSSION

When Xenopus sperm nuclei are added to egg extracts, there is a ∼20 min prereplicative lag period during which chromatin decondenses, origins are licensed and a nuclear envelope reforms. S phase only starts when these steps are completed. The results reported here demonstrate that (i) no topo II activity is required during S phase to promote a normal rate of DNA replication; (ii) trapping topo IIα-DNA clamps by ICRF-193 during nuclear assembly, but not later, slows down the activation of origin clusters and the progression of replication forks in a checkpoint-independent manner; (iii) topo IIα acts in an ICRF-193-resistant manner during nuclear assembly to negatively regulate MCM loading and to establish a temporal program of origin cluster activation during the subsequent S phase.

### Topo IIα dispensability during S phase

The conclusion that topo II is not required during S phase for a normal rate of DNA replication is based on two observations. First, the topo II catalytic inhibitor ICRF-193 had no detectable effect on replication initiation or elongation when it was only present during S phase. This result is in apparent contrast with an earlier study reporting that addition of 20 µM ICRF-193 after replication initiation slowed replication by ∼10% (45). We repeated this experiment six times using 100 µM ICRF-193 and found that on average, replication was not inhibited (Figure 1F). When the data points of the single experiment reported in (45) were plotted along those of Figure 1F, they fell within the same cloud of points, and the regression and correlation coefficients were practically unchanged (Supplementary Figure S5). We conclude that the data of the two studies are consistent and that when multiple experiments are averaged no significant inhibition of DNA replication is observed when ICRF-193 is only present during S phase. This result cannot be explained by a failure to inhibit topo II within replicating nuclei because ICRF-193 was observed to alter chromatin structure in these conditions (36) and to block decatenation of replicating DNA in egg extract equally efficiently when added at 0 or 20 min (Supplementary Figure S2). Second, immunodepletion of topo IIα, the sole topo II isozyme expressed in Xenopus eggs, did not slow down but instead accelerated the replication of sperm nuclei with respect to mock depletion. These experiments demonstrate that assembly and elongation of replication forks do not require topo II activity, in agreement with studies showing that yeast cells harboring a thermosensitive or a degron mutant topo II complete DNA replication with normal kinetics (51–54).

These observations imply that the excess of parental DNA strand linkage [(+)ΔLk] generated by the absence of topo II activity does not impair replication fork progression. This suggests that if the (+)ΔLk takes the form of (+) supercoils in front of the forks, topo I removes them so efficiently that fork progression is not affected. Alternatively, the (+)ΔLk may take the form of (+) precatenanes behind the forks so that the torsional stress is relieved ahead of the forks. Study of plasmid DNA replication in Xenopus egg extracts favors the latter possibility because replicative intermediates generated in the presence of ICRF-193 do have a detectably increased ΔLk and because etoposide traps topo IIα behind but not in front of the forks (33,34).

### ICRF-193 action before and during S phase

The experiments presented here clarify the mechanism by which ICRF-193 slows down S phase (27). ICRF-193 inhibited both origin cluster activation and replication fork progression when added during but not after nuclear assembly. As topo IIα immunodepletion did not slow S phase, catalytic inhibition of topo IIα cannot be the mechanism for the replication-slowing effects of ICRF-193, which instead must be caused by the trapping of topo IIα-DNA clamps.

Then, why do these clamps inhibit replication when they form during but not after nuclear assembly? We considered that the clamps could perturb some prereplicative process required for optimal S phase progression such as origin licensing or chromatin modification. However, we did not detect a consistent effect of ICRF-193 on origin licensing. ICRF-193 neither reduced the binding of factors involved in origin activation such as RecQ4 and Cdc45. We noticed that nuclear-envelope-dependent chromatin decondensation associated with S phase progression was specifically perturbed by prereplicative addition of ICRF-193. However, topo IIα depletion similarly perturbed chromatin decondensation without slowing S phase progression, suggesting that this decondensation process is not rate-limiting for replication. We therefore suggest that topo IIα clamps trapped during nuclear assembly perturb S phase by acting as replication roadblocks, and that clamps trapped after nuclear assembly fail to do so because topo IIα is relocated from unreplicated DNA to replicated sister chromatids during S phase. First, topo IIα dissociated from unreplicated DNA during nuclear assembly. Second, topo II was reported to specifically bind replication origins at the start of S phase in budding yeast (55). Third, topo IIα specifically interacts with PCNA, a protein complex located behind the forks (56). Fourth, cleaved topo IIα-DNA complexes trapped by etoposide are located behind but not in front of forks in Xenopus egg extracts (33). Fifth, DNA replication allows topo IIα to become tightly associated with sister chromatids in Xenopus egg extracts (50). Therefore, although there is no direct evidence for roadblocking, relocalization of topo IIα can simply explain the time-of-addition effects of ICRF-193 if topo IIα clamps act by forming roadblocks to the replicative helicase. The reduction in fork density measured by DNA combing in the presence of ICRF-193 added at 0 min, despite the normal or increased chromatin binding of Cdc45, an activator of the MCM2-7 helicase, suggests that topo IIα clamps trapped on unreplicated DNA reduce the efficiency of origin unwinding after Cdc45 recruitment.

Our experiments suggest that although ICRF-193 slows down S phase, it does not create a major block to replication termination. Consistently, when plasmid DNA was replicated in egg extracts in the presence of ICRF-193, covalently closed hypercatenated dimers but no late replicative intermediates accumulated, demonstrating that decatenation but not termination was blocked (33,34). In contrast to these results, it was reported that ICRF-193 does not significantly affect the initiation of DNA replication and only inhibits the completion of DNA replication when Xenopus sperm nuclei are replicated in egg extracts (45). We believe that these conclusions are incorrect. First, the DNA combing data in (45) showing that ICRF-193 did not change the distribution of eye-to-eye distances and the proportion of unreplicated gaps >15 kb within active origin clusters are entirely consistent with our data and only confirm that once a cluster is active, ICRF-193 does not perturb initiation within it. In our experiments, however, we demonstrated that the global fork density was lower in the presence of ICRF-193 owing to a slower activation of origin clusters. This key observation was missed in (45) because non-replicated DNA fibers were not taken into account. Second, the [α-^32^P]dATP incorporation curves presented in (45) show that ICRF-193 slowed replication through most of S phase rather than just at the end of S phase, in agreement with our observations and with an earlier study (27) (see also Supplementary Figure S5). Therefore, slower origin cluster activation and slower fork progression can explain better than a specific termination block why replication was slower in the presence of ICRF-193. The last argument for a ‘new discrete termination step blocked by ICRF’ in (45) is a DNA combing experiment in which non-terminated BrdU-labeled replicons present in nuclei incubated for 120 min in egg extract ± ICRF-193, resumed elongation in the presence of dig-dUTP after transfer in fresh extract without ICRF-193. It was found that the number of incorporation sites was increased 4-fold when the first extract contained ICRF-193. However, this is not surprising, given that replication at 120 min was less advanced with ICRF-193 than in the control (75–83% versus 91–97%). As the effect of ICRF-193 on dig-dUTP incorporation in the second extract was not tested, it is unclear whether this represented the execution of an ICRF-193-sensitive termination step rather than the trivial completion of slower initiated and elongated replicons. Finally, these authors failed to discuss our previous observation that decatenation, but not termination, was blocked when plasmid DNA was replicated in egg extracts in the presence of ICRF-193 (33). Although we cannot formally exclude that ICRF-193 perturbs termination when sperm chromatin but not plasmid DNA is the template, ICRF-193 did not cause a prolonged accumulation of replicon-sized nascent strands in our experiments with sperm nuclei (Figure 3D). The delayed completion of replication in the presence of ICRF-193 can therefore be explained by slower initiation and elongation with no need to invoke a specific block to termination.

The replicative stress generated by the topo IIα clamps, if any, does not appear to reach the threshold required to activate the replication checkpoint. Topo II clamps trapped by ICRF-193 can freely slide on double-stranded DNA (35). Although the replicative helicase might slow down when encountering a topo IIα clamp, this is not predicted to uncouple polymerase from helicase progression and to generate sufficient single-stranded DNA to activate the checkpoint. This is consistent with the lack of detectable Chk1 or RPA phosphorylation following ICRF-193 addition to Xenopus egg extracts (45), although it is controversial whether Chk1 is a downstream effector of the replication checkpoint in Xenopus (46,47).

### Topo IIα regulates MCM loading and origin cluster firing time

The experiments described here demonstrate a novel prereplicative role for topo IIα in regulating MCM loading and establishing origin cluster firing time. First, topo IIα rapidly bound to the decondensing sperm chromatin then progressively dissociated during origin licensing. Second, topo IIα depletion increased MCM loading ∼2-fold, increased RecQ binding and advanced the firing time of origin clusters, without affecting initiation and fork progression within clusters. The resulting acceleration of S phase was negated by adding back recombinant topo IIα. Third, addition of recombinant topo IIα to undepleted extracts before but not after origin licensing reduced MCM loading and RecQ binding and slowed S phase progression ∼2-fold.

We considered that topo IIα might regulate origin licensing by controlling DNA topology. DNA topology has been reported to significantly influence the binding of metazoan ORC to DNA (57). However, as ICRF-193 did not affect origin licensing, the regulation of this process by topo IIα does not seem to require its catalytic activity. A potential limitation to this conclusion is that ICRF-193 only inhibits topo II after a first round of strand-passing activity (58). We cannot exclude that first-turnover events are sufficient for topo IIα to regulate licensing. Nevertheless, the fact that exogenous topo IIα addition enhances the effect of endogenous topo IIα is more in agreement with a structural role of topo IIα in origin licensing regulation. Topo IIα has long been proposed to have additional functions to its strand-passing activity. Early studies suggested that topo IIα acts as a ‘DNA loop fastener’ (59,60) and has a structural role in mitotic chromosome structure (61). Yeast topo II can stably bind DNA crossovers in a way that is not directly related to its catalysis of DNA transport (62), binds origins at the start of S phase and functionally interacts with Hmo1, an HMG protein also able to bind four-way DNA junctions, to maintain genome stability during S phase (63). In human cells, topo II interacts with ORC in G1 phase at the lamin B2 origin (64), and HMGA1a can recruit ORC to heterochromatic regions (65). The ability of topo IIα and HMG proteins to bind each other and/or ORC and/or DNA crossovers independently of strand-passing events may underlie origin clustering and licensing regulation.

A recent model for replication timing regulation in yeast is based on the observation that multiple MCMs are loaded at each origin. In this model, each MCM has a low probability of firing but origins that have more MCMs loaded will be more efficient and therefore show an earlier and sharper firing time distribution (11). In Xenopus, it is unclear whether MCM loading is homogeneous or heterogeneous through the genome. Furthermore, the target for replication timing regulation is the origin cluster rather than the individual origin. Nevertheless, the first initiation event within a cluster may be the rate-limiting step controlled by MCM density. One possible scenario is that topo IIα restrains MCMs loading at only some clusters to program them for late replication, and that topo IIα depletion abolished (and its addition enhanced) this intercluster difference in MCM loading and firing time. An alternative and simpler scenario is that MCM loading is homogeneous over the genome, and that the spreading of cluster firing time is only a stochastic effect. In this case, topo IIα depletion or addition would respectively increase or decrease the overall MCM density and sharpen or broaden the spreading of cluster firing time, consistent with our observations.

Our observation that manipulating topo IIα levels affects RecQ4 binding in a correlated manner to MCM loading is consistent with MCM level controlling the recruitment of downstream replication factors. However, we do not exclude the possibility that topo IIα levels regulate chromatin accessibility to RecQ4 and other replication factors independently of, and in addition to, MCM loading, to spread the firing time of origin clusters.

The evidence reported here for a novel function of topo IIα in regulating origin cluster activation extends previous evidence for its role in replicon programming. It was reported that Xenopus erythrocyte nuclei replicate inefficiently in interphasic egg extracts but replicate efficiently if they are first incubated in a mitotic extract, which shortens the replicon size in an ICRF-193-sensitive, topo IIα-dependent manner (66). The novel function of topo IIα reported here is not mitotic but interphasic. It does not affect replicon size but restrains MCM loading and spreads the activation time of origin clusters in an ICRF-193-resistant manner. These results are consistent with reports showing that replication timing is established during the G1 phase in yeast and mammalian cells and is an independent process of origin specification (3,67) and identify topo IIα as a key regulator of the replication timing program and S phase length in Xenopus. It would be interesting to investigate whether the timing and origin decision points are sensitive to topo II depletion or inhibition in other organisms.

Topo II is the target of many anticancer drugs (68). Compounds closely related to ICRF-193 are used in clinical practice to reduce the cardiac cytotoxicity of anthracyclines (69,70). Perturbations of the replication program reported here may occur during clinical use of topo II inhibitors and may contribute to genome damage and cellular toxicity.

## SUPPLEMENTARY DATA

Supplementary data are available at NAR Online: Supplementary Figures 1–6.

Supplementary Data
