# Supplementary material for: DNA topoisomerase IIα controls replication origin cluster licensing and firing time in Xenopus egg extracts
Source: Nucleic Acids Res. 2013 Jun 11;41(15):7313–31. doi: 10.1093/nar/gkt494 (PMC3753627; doi:10.1093/nar/gkt494)
Supplement: Supplementary Data [file supp_41_15_7313__index.html]

DNA topoisomerase IIα controls replication origin cluster licensing and firing time in Xenopus egg extracts — DNA topoisomerase IIα controls replication origin cluster licensing and firing time in Xenopus egg extracts — Supplementary Data 

# DNA topoisomerase IIα controls replication origin cluster licensing and firing time in Xenopus egg extracts

## Supplementary Data

files

**Files in this Data Supplement:**

- Supplementary Data - pdf file
