# Supplementary material for: DNA topoisomerase IIα controls replication origin cluster licensing and firing time in Xenopus egg extracts
Source: Nucleic Acids Res. 2013 Jun 11;41(15):7313–31. doi: 10.1093/nar/gkt494 (PMC3753627; doi:10.1093/nar/gkt494)
Supplement: Supplementary Data [file supp_gkt494_nar-02454-v-2012-File010.pdf]

Supplementary Material to:

DNA topoisomerase IIα controls replication origin cluster licensing and firing time in Xenopus egg extracts

Vincent Gaggioli[1#], Barbara Le Viet[1#], Thomas Germe[1] and Olivier Hyrien[1*]

[1]Institut de Biologie de l'Ecole Normale Supérieure (IBENS), CNRS UMR8197, Inserm U1024, 46 rue d'Ulm, 75005 Paris, France
*To whom correspondence should be addressed. Tel: + 33 1 44 32 39 20; Fax: + 33 11 44 32 35 70; Email: hyrien@biologie.ens.fr
[#]Equal first authors

Contents:

Figure S1

Figure S2

Figure S3

Figure S4

Figure S5

Figure S6

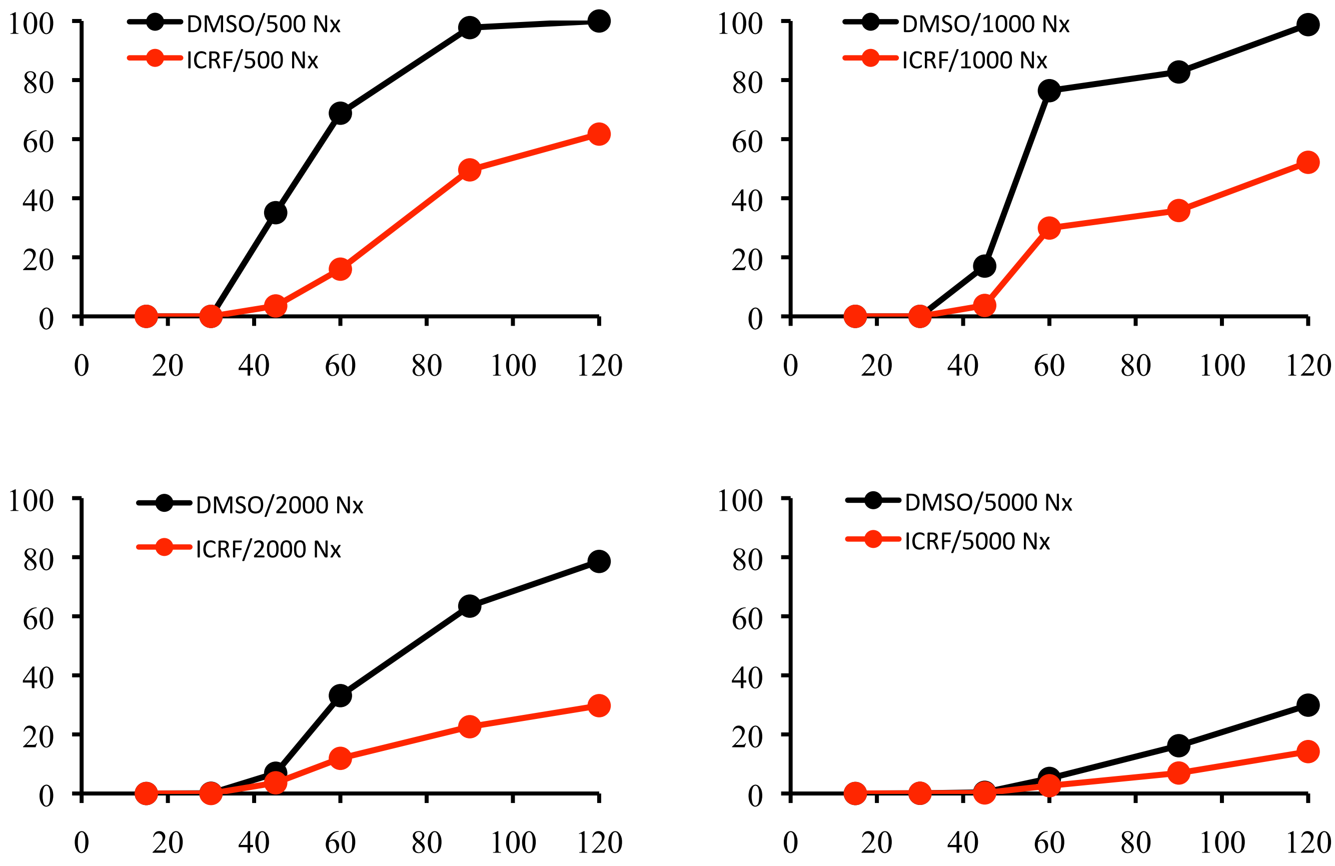

Figure S1. Kinetics of DNA replication at different concentrations of sperm nuclei in Xenopus egg extracts. Sperm nuclei were replicated at 500, 1000, 2000 or 5000 nuclei/μL in egg extract in the presence of DMSO or 100 μM ICRF-193 and replication was monitored by [a^{32}P]-dATP incorporation as described in Materials and Methods. Replication started later and progressed at a slower rate as the concentration of nuclei was increased. ICRF-193 reduced the rate of replication ~2-fold at all nuclei concentrations.

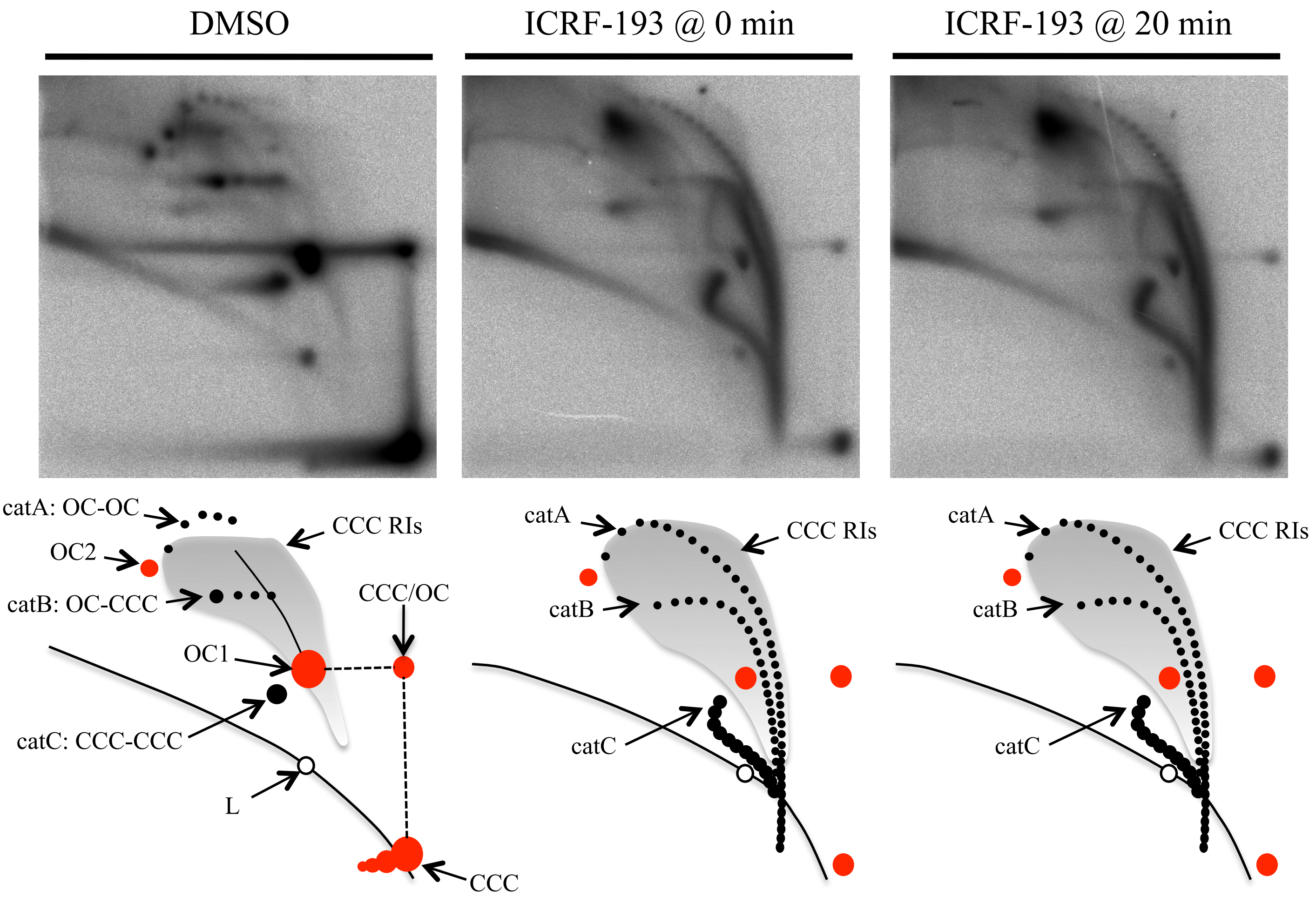

Figure S2. Topological analysis of plasmid DNA replication in Xenopus egg extracts and effect of ICRF-193 added at 0 min or at 20 min. pBR322 DNA was incubated in an egg extract in the presence of [a^{32}P]-dATP and in the presence or absence of 100 μM ICRF-193 added at 0 min or at 20 min and analysed by 2D-gel electrophoresis as described (33,34). In the DMSO (no drug) control, most replication products weres open circular (OC), covalently closed circular (CCC) and linear (L) plasmid monomers. The CCC/OC spot consists of molecules that migrated as CCC in the first dimension, were subsequently nicked, and migrated as OC in the second dimension. A small amount of A-type (OC-OC), B-type (OC-CCC) and C-type (CCC-CCC) catenated dimers was also observed. CCC replication intermediates (CCC RIs) migrated as a broad smear as described (33,34). In the presence of ICRF-193 (added at 0 min or 20 min) very few monomers were produced. Most replication products appeared as A-, B- and C-type catenated dimers with a much higher and broader node number distribution than in the control. The electrophoretic migration of CCC RIs changed due to their increased ΔLk as described earlier (33,34). The experiment shows that ICRF-193 blocked decatenation of the replicating plasmid as effectively when added at 20 min as at 0 min.

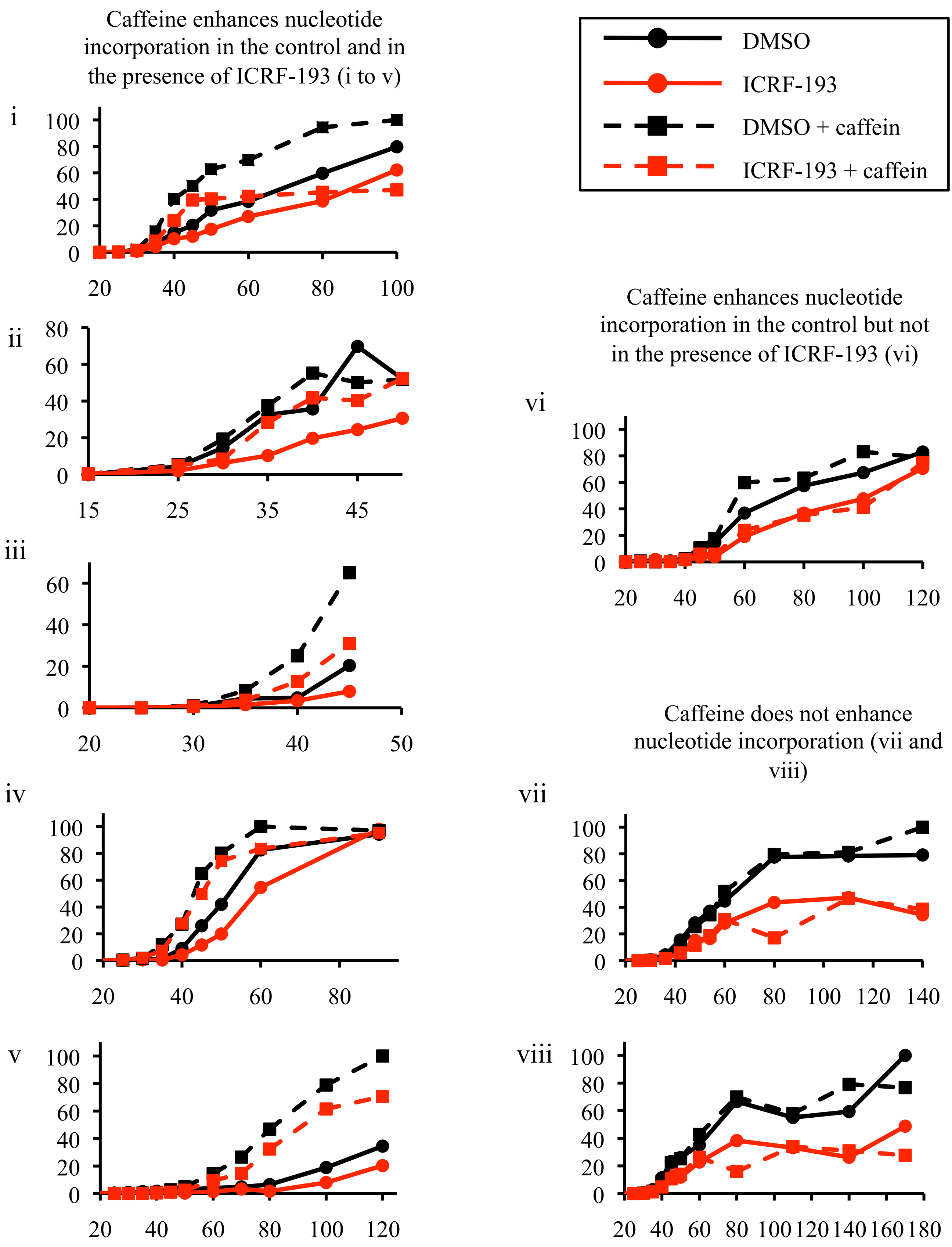

Figure S3. Effect of caffeine on the ICRF-193-induced inhibition of replication in 8 independently prepared egg extracts. Sperm nuclei were incubated in eight independently prepared egg extracts containing [α-^{32}P]dATP plus or minus 100µM ICRF-193 and/or 5mM caffeine. The extent of DNA replication was measured at the indicated time points. ICRF-193-induced inhibition of replication was partially rescued by caffeine in extracts (i to v) but was not rescued in extracts (vi to viii). Note that in all eight extracts the ICRF-193 + caffeine curve remained below the DMSO+caffeine curve.

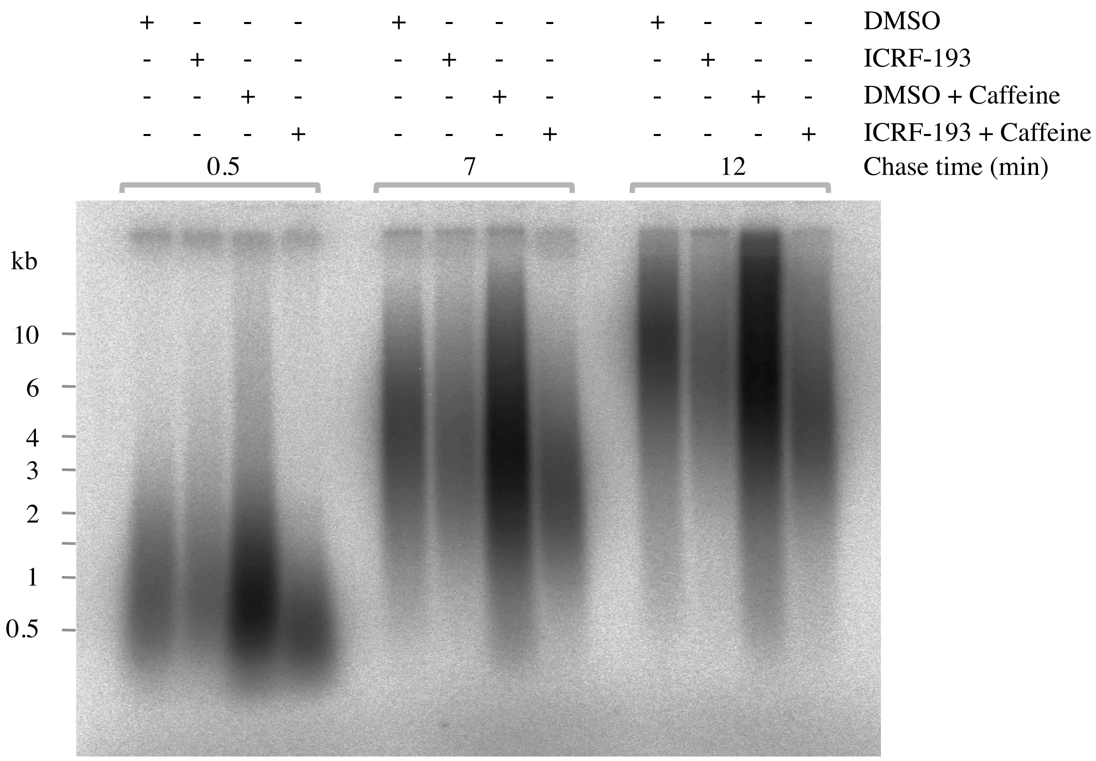

Figure S4. Caffeine does not affect the slower growth of nascent strands in the presence of ICRF-193. Sperm nuclei incubated in egg extract plus or minus ICRF-193 and/or caffeine were labeled at 28 min with a 2 min pulse of [α-^{32}P] dATP and chased for the indicated times with unlabeled dATP in the presence of roscovitin. Nascent strand abundance and growth was monitored by alkaline gel electrophoresis.

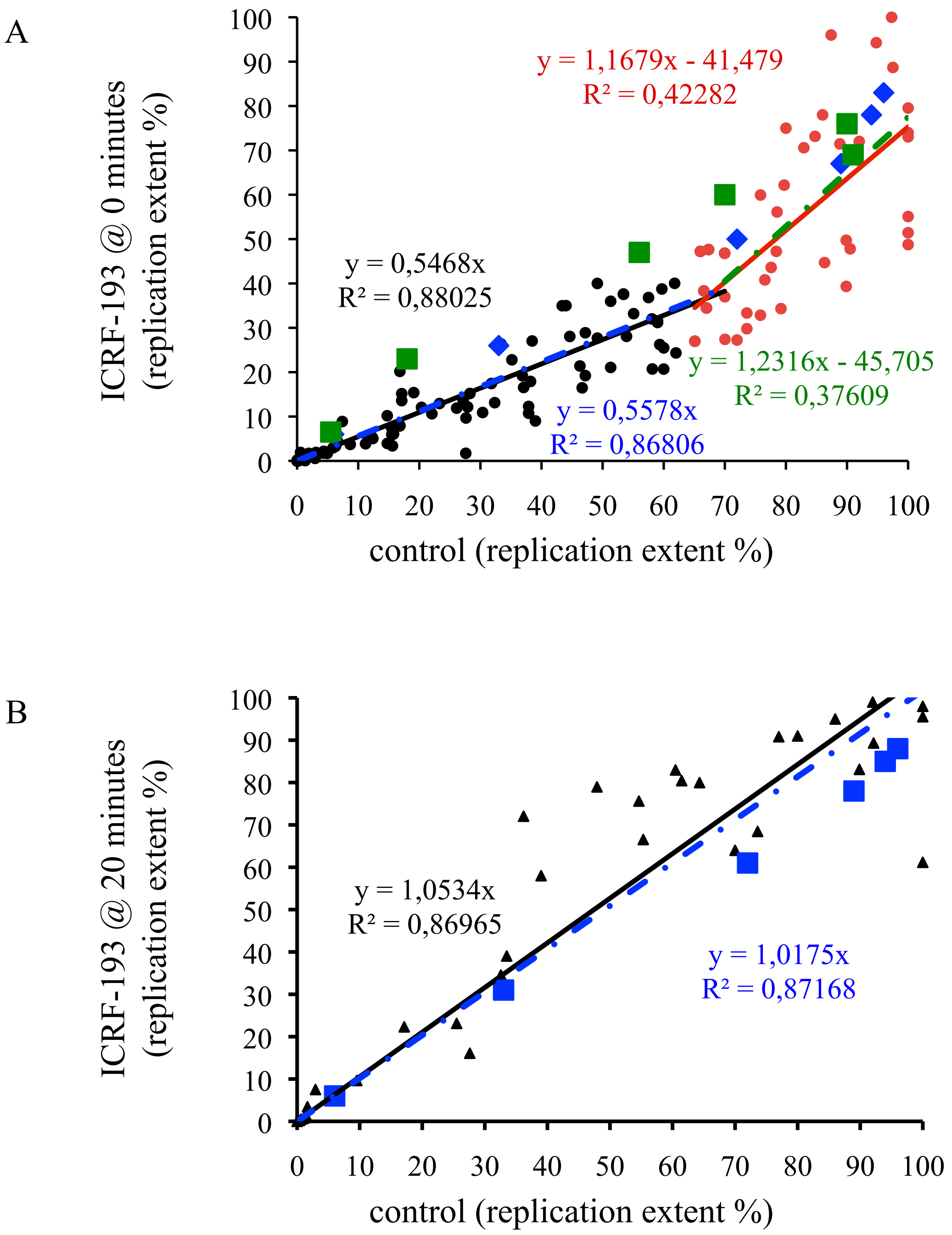

Figure S5. Consistency of the [α-^{32}P]dATP incorporation experiments reported in (45) and in our study. (A) Percentage of replication in the presence of ICRF-193 added at 0 min plotted against the control. The blue diamonds and green squares are from the two experiments reported in Figure 5A and Figure S6A, respectively, of reference (45) using 20 μM ICRF-193. The dark and red dots are from our Figure 1E using 100 μM ICRF-193. (B) Percentage of replication in the presence of ICRF-193 added at 20 min (this study) or 45 min (reference (45)) plotted against the control. The blue squares are from the experiment reported in Figure 5A of reference (45) using 20 μM ICRF-193. The dark triangles are from our Figure 1E using 100 μM ICRF-193. In both (A) and (B), the solid lines are fits to the data of Figure 1E alone and the dotted lines are fits to the pooled data of (45) and Figure 1E. The Figure shows that in both the (A) and (B) cases the data of (45) collapse with ours.

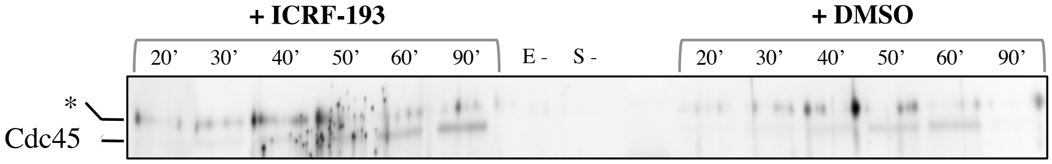

Figure S6. Effect of topo IIα inhibition by ICRF-193 on chromatin binding of Cdc45. Sperm nuclei were incubated in undepleted extracts added with DMSO or 100µM ICRF-193 at 0 min. Purified chromatin at indicated times was analysed by western blotting for Cdc45 protein. Samples without egg extract (E-) or without sperm nuclei (S-) were loaded as internal controls. The asterisk (*) indicates a cross-reacting band. Cdc45 binding was detected at 40-60 min in the DMSO control. ICRF-193 did not detectably affect the binding of Cdc45 at 40-60 min but prolonged it until 90 min, consistent with the slower S phase observed in the presence of the drug.
